# Development of a Web-Based and Mobile App to Support Physical Activity in Individuals With Rheumatoid Arthritis: Results From the Second Step of a Co-Design Process

**DOI:** 10.2196/resprot.3795

**Published:** 2015-02-09

**Authors:** Åsa Revenäs, Christina H Opava, Cathrin Martin, Ingrid Demmelmaier, Christina Keller, Pernilla Åsenlöf

**Affiliations:** ^1^Division of PhysiotherapyDepartment of Neurobiology, Care Sciences and SocietyKarolinska InstitutetHuddingeSweden; ^2^Department of RheumatologyKarolinska University HospitalStockholmSweden; ^3^PhysiotherapyDepartment of NeuroscienceUppsala UniversityUppsalaSweden; ^4^InformaticsJönköping International Business SchoolJönköpingSweden

**Keywords:** eHealth, Internet intervention, physical activity, rheumatoid arthritis, behavior change techniques, participatory design

## Abstract

**Background:**

Long-term adherence to physical activity recommendations remains challenging for most individuals with rheumatoid arthritis (RA) despite evidence for its health benefits.

**Objective:**

The aim of this study was to provide basic data on system requirement specifications for a Web-based and mobile app to self-manage physical activity. More specifically, we explored the target user group, features of the future app, and correlations between the system requirements and the established behavior change techniques (BCTs).

**Methods:**

We used a participatory action research design. Qualitative data were collected using multiple methods in four workshops. Participants were 5 individuals with RA, a clinical physiotherapist, an officer from the Swedish Rheumatism Association, a Web designer, and 2 physiotherapy researchers. A taxonomy was used to determine the degree of correlation between the system requirements and established BCTs.

**Results:**

Participants agreed that the future Web-based and mobile app should be based on two major components important for maintaining physical activity: (1) a calendar feature for goal setting, planning, and recording of physical activity performance and progress, and (2) a small community feature for positive feedback and support from peers. All system requirements correlated with established BCTs, which were coded as 24 different BCTs.

**Conclusions:**

To our knowledge, this study is the first to involve individuals with RA as co-designers, in collaboration with clinicians, researchers, and Web designers, to produce basic data to generate system requirement specifications for an eHealth service. The system requirements correlated to the BCTs, making specifications of content and future evaluation of effectiveness possible.

## Introduction

There is solid evidence of the benefits of physical activity (PA) in individuals with rheumatoid arthritis (RA), for example, improved functional capacity and decreased risk of cardiovascular disease [1-3]. Despite these apparent benefits, only 11% of individuals with RA report pursuing healthy PA regimens based on recommendations [4]. Integration of PA into everyday life remains a challenge, even with the development of interventions targeting the specific needs of this population [2,3,5-8]. Consequently, new strategies are required to achieve long-term adherence to PA recommendations.

The challenges are partly due to PA being a complex behavior determined by a number of bio-psychosocial factors [9-11]. Social cognitive theory (SCT) [12] has helped identify factors important for health behavior change in chronic conditions, such as the importance of self-regulation skills to facilitate PA in individuals with RA [13]. This theory has also been recommended to inform self-management interventions in arthritis to increase its effects [14].

Another mechanism of increasing the impact and scope of PA interventions is using the Internet. The Internet has the potential to reach large populations [15,16] and is convenient and readily accessible [17]. To the best of our knowledge, only one previous study has evaluated the effects of an Internet-supported PA intervention for individuals with RA [7]. However, the results showed that only minor integration of PA into participants’ everyday life was achieved [18]. In addition, this intervention did not explicitly incorporate behavior change techniques (BCTs) to self-manage PA [7].

A BCT is defined as the active component (eg, goal-setting, self-monitoring) of a behavioral intervention that alters or redirects the target behavior [19]. Self-monitoring with at least one additional BCT has been reported to increase the efficacy of PA interventions [20]. The use of a BCT taxonomy in the development of a PA intervention may improve reports on the content of the intervention, help identify effective intervention components, and facilitate comparison of results between studies.

A new strategy used to develop interventions is participatory design [21]. This method has frequently been applied to the organizational development and design of health information systems [22,23]. Participatory design allows users to be actively involved as co-designers [21,24]. More recently, participatory design has been used to design Internet-based health care services for multiple purposes and target groups [25-28]. We have not been able to identify any previous study involving individuals with RA in the development of an Internet service to support the self-management of PA.

In this project, we co-design a Web-based and mobile app for the self-management of physical activity in patients with rheumatoid arthritis. During the first step of the co-design process, focus-group participants diagnosed with RA presented ideas on core features that would be important to consider when developing the app. These features included content, customizable options, the user interface, and access and implementation [29].

This report presents results from the second step of the co-design process. The overall aim was to provide basic data on system requirement specifications for a Web-based and mobile app for PA self-management in individuals with RA. Specific objectives included exploring the characteristics of the future target-user group, the features of the future app, and the correlation between established BCTs and system requirements, that is, what the app should provide, arrange, or do.

## Methods

### Design

To involve the participants as co-designers, we applied a participatory action research design [30]. Data were collected during and in between four workshops in February and March 2013 at Uppsala University, Sweden. We used multiple methods for data collection and analysis to capture dimensions of participants’ proposals, preferences, and agreements. This study was approved by the regional ethical review board in Stockholm (D.nr. 2010/1101-31/5).

### Selection of Participants

A sensibly sized co-design group (N=10) was formed to capture different perspectives. We included 5 adults diagnosed with RA, a clinical physiotherapist, an officer from the Swedish Rheumatism Association (SRA), a Web designer, and 2 physiotherapy researchers (ID, CO) with knowledge of the theory of health behavior change and evidence-based knowledge on PA in RA. The inclusion criteria were individuals with adequate communication skills in Swedish who had access to the Internet and were comfortable using it. In addition, we wanted the participants with RA to reflect the diversity of the population of RA regarding age, gender, years with diagnosed RA, and PA habits.

To ensure the participants had the required knowledge, all participants were identified through our research and clinical network. The first author (ÅR) contacted them by email, and those who provided preliminary consent received verbal information about the study. Written information and a questionnaire on background characteristics, expertise, PA behavior, and Internet habits were then disseminated. Participants provided their final consent for participation by attending the first workshop. Tables 1 and 2 present the participants’ characteristics, expertise, and experiences.

In addition to the participants, 4 researchers were present during the workshops. Of these researchers, one acted as a video camera operator (CM, with experience in qualitative, video-based research and group interaction), one had overall responsibility for alignment of the process (PÅ, with expertise in behavioral medicine), and 2 of them acted as non-participatory observers (CK, with expertise in health informatics; ÅR, with expertise in musculoskeletal disorders). A moderator (with expertise in the design of Web-based and mobile apps and experience of group moderation) facilitated the entire process during and between the workshops.

**Table 1:** Demographics of participants (N=10).

Participants	Age, year	Gender	Children <18 years	Living with other adults	Years since RA diagnosis	Other chronic disease	Education & work experience
1. RA	73	Male	No	Yes	>10	No	High school
2. RA	69	Female	No	Yes	>10	No	University, PA/Exercise, Health care, Research, Exercise group leader
3. RA	69	Female	No	No	>10	No	University <3 years
4. RA	43	Female	Yes	Yes	>10	Yes	University <3 years
5. RA	34	Female	Yes	Yes	5-10	No	University, Health care, Research
6. Clinical PT^a^	49	Female	Yes	Yes	No	No	University, PA/Exercises, Health promotion, Health care, Research, Exercise group leader
7. SRA^b^	46	Male	No	No	No	Yes	University, Product development, Management
8. Web designer	57	Male	No	Yes	No	No	University <3 years, PA/Exercise, Computer programing, Product development, Management, Health promotion
9. Researcher	59	Female	No	Yes	>10	Yes	University, PA/Exercise, Health care, Research, Management
10. Researcher	53	Female	No	Yes	No	No	University, PA/Exercise, Health care, Research, Management, Exercise group leader, Health promotion

^a^Physiotherapist

^b^Swedish Rheumatism Association

**Table 2:** Computer, mobile phone, and PA experiences of participants (N=10).

Participants	Internet use	Uses/have tried PA apps	Own a mobile phone	Meet PA recommendations aerobic exercise 30 min ≥5 days/week^a^	Meet PA recommendations strength training ≥2 days/week^a^
RA	Several times/day	No	No	Precontemplation phase	Maintenance phase
RA	Several times/day	No	Yes	Maintenance phase	Maintenance phase
RA	Several times/day	No	No	Used to	Precontemplation phase
RA	Several times/day	No	Yes	Maintenance phase	Maintenance phase
RA	Several times/day	Yes	Yes	Action phase	Preparation phase
Clinical PT^b^	Once/ week	No	No	Maintenance phase	Used to
SRA^c^	Several times/day	Yes	Yes	Preparation phase	Preparation phase
Web designer	Several times/day	Yes	Yes	Maintenance phase	Maintenance phase
Researcher	Several times/day	Yes	Yes	Action phase	Contemplation phase
Researcher	Several times/day	No	Yes	Maintenance phase	Preparation phase

^a^Physical activity habits were assessed with the question “Do you follow the recommendations for health enhancing physical activity, as described above?”, with the possible answers “maintenance phase” (has followed the recommendations for at least 6 months), “action phase” (has followed the recommendations less than 6 months), “preparation phase” (plan to follow the recommendations within 1 month), “contemplation phase” (plan to followed the recommendations within 6 months), “precontemplation phase” (do not plan to followed the recommendations within 6 months), used to (used to be physically active but have been less physically active the previous months).

^b^Physiotherapist

^c^Swedish Rheumatism Association

### The Workshops

The four workshops occurred at intervals of 1-4 weeks. They took place in university lecture rooms with interactive boards (SMART boards). The first workshop began by presenting the aim, overall content, procedure of the workshops, and the participants’ different roles and expertise during the workshops. Discussion started with the results from the first step of the co-design process, that is, core features of the future app based on the outcomes of the previous focus-group interviews [29]. Three authors (ÅR, CM, and PÅ), the Web designer, and the moderator planned each workshop iteratively such that each workshop was built on the results and experiences from the preceding workshop.

Table 3 presents an overview of the major content of and participants at each workshop. Even though the participants had ensured they could be present at all four workshops, some of the participants had to cancel due to unplanned life events such as sickness.

A pilot workshop was held prior to the start of the study, with the aim of testing data collection procedures, for example, technical solutions for the video recordings, the interactive board, and the feasibility of the observation protocols.

**Table 3:** Overview of the four workshops with a description of major content and attending participants.

Workshop	Major content
Workshop 1: Brainstorming (P^a^=1,2,5,6,7,8,9,10)	Introduction
	Warm-up session
	Brainstorming on needs and proposed features
Workshop 2: Brainstorming and narrowing (P=1,2,3,4,5,6,7,8,9,10)	Warm-up session
	Transforming the participants needs to proposed feature
	Creation of the first preliminary framework of the app
	Focus group interviews^b^
Workshop 3: Specification of features on the app (P=1,2,3,4,6,7,8,9)	Presentation of available PA apps
	Presentation of the first preliminary framework presented as a mobile phone app
	Creation of the second preliminary framework of the app
Workshop 4: Specification of features on the app (P=1,2,3,5,6,7,8,9)	Presentation of the second preliminary framework presented as a mobile phone app
	Continuous specification of features
	Focus group interview^b^

^a^The participants attending the workshop.

^b^Focus group interviews were performed to explore the participants’ experiences and perceptions of co-design. These data will be analyzed and presented elsewhere.

### Data Collection

Data were collected during the workshops using (1) an online notice board (Trello), (2) interactive boards, (3) Post-it notes grouped on plastic sheets, (4) video recordings, and (5) observation protocols. Postings on the online notice board were also collected between the workshops. The interactive boards and Post-it notes grouped on plastic sheets were used to produce preliminary frameworks, that is, outlines of the arrangement of contents and functionalities for the future app, which provided “visual guides” on how the workshop participants’ proposed features could be arranged on a future Web-based and mobile app.

In addition, focus-group interviews were performed immediately after the second and fourth workshops to explore the participants’ experiences and perceptions of being involved in the co-design. These data will be analyzed and presented elsewhere.

### Data Management and Analysis

Between each workshop, the first author (ÅR) mapped out and outlined the video recordings and compiled the observation protocols from the 2 non-participatory observers. Additionally, the preliminary frameworks produced on the interactive board and the plastic sheets were programmed by the moderator as mobile phone apps to be presented at the following workshop.

After the final workshop, 3 authors (ÅR, PÅ, and ID) analyzed the data by triangulating the different data-collection methods. The main analysis was based on the postings on the online notice board and the preliminary frameworks (Table 4). These data were clarified and validated by use of the data from the observation protocols, the video recordings, the Post-it notes on outcome measures, and the programmed mobile phone apps (Table 5).

**Table 4:** Overview of the data collection for main analysis.

Data	Postings on the online notice board	First preliminary framework produced on the interactive board	Second preliminary framework produced on a plastic sheet with grouped Post-it notes
Characteristics of the target user group	X		
Features	X	X	X

**Table 5:** Overview of the data collection for clarification and validation.

Data	Observation protocols compiled from 2 non-participatory observers	Video recordings	Post-it notes grouped on plastic sheets on PA performance and health outcome measures	Preliminary frameworks programmed as a mobile phone app
Characteristics of the target user group	X	X		
Features	X	X	X	X

Data on the characteristics of the target-user group, that is, the users of the future app, were retrieved from a list compiled on the online notice board (ÅR). These data were clarified and validated by examining sequences on the video recordings where the issue was discussed. The observation protocols helped to identify these sequences.

The features and system requirements were analyzed by one author (ÅR). Data on features were retrieved from the online notice board and the first and second preliminary frameworks. First, the data from the online notice board were condensed and grouped into clusters reflecting similar core meanings. The clusters were then reformulated to system requirements, that is, what the app should provide, arrange, or do. Each system requirement was then correlated with features in the first and second preliminary frameworks. In the latter workshops, participants agreed to combine these two preliminary frameworks, which resulted in the first version of the app. Again, the process was validated and complemented with additional data from selected video sequences identified in the observation protocols.

Next, the system requirements were coded based on established BCTs using a taxonomy [19]. The taxonomy consists of 93 BCTs clustered into 16 hierarchical groups (1-16); techniques with similar active components are grouped together. Each technique was given a code (eg, 1.1, 1.2, 2.3) and labeled, defined, and exemplified. The first author (ÅR) coded the system requirements. The fourth author (ID) reviewed the coding, and disparities were discussed. Following the third iterative cycle of reviewing and discussing the coding, the 2 authors (ÅR and ID) agreed upon the coding. The last author (PÅ) was then introduced to the analysis, resulting in one further revision of the coding, the clustering of features, the wording of the system requirements and their correlation with features on the app.

## Results

### The App

Participants agreed that the app should be based on two preliminary frameworks: (1) “My self-monitoring”: a calendar feature for planning, setting goals, and recording PAs and progress, and (2) “My peer group”: a small community feature for positive feedback and support from peers. The app should support the maintenance of PA and was proposed as being a lifelong companion that should encourage PA during good and bad disease periods. As one of the participants expressed, “I want to be able to use the app even during periods when I do my exercises and feel healthy”*.* The proposed name of the app was “tRAppen,” which correspond to “stairs” in English and derives from terms related to PA, RA, and app in Swedish.

**Figure 1:**
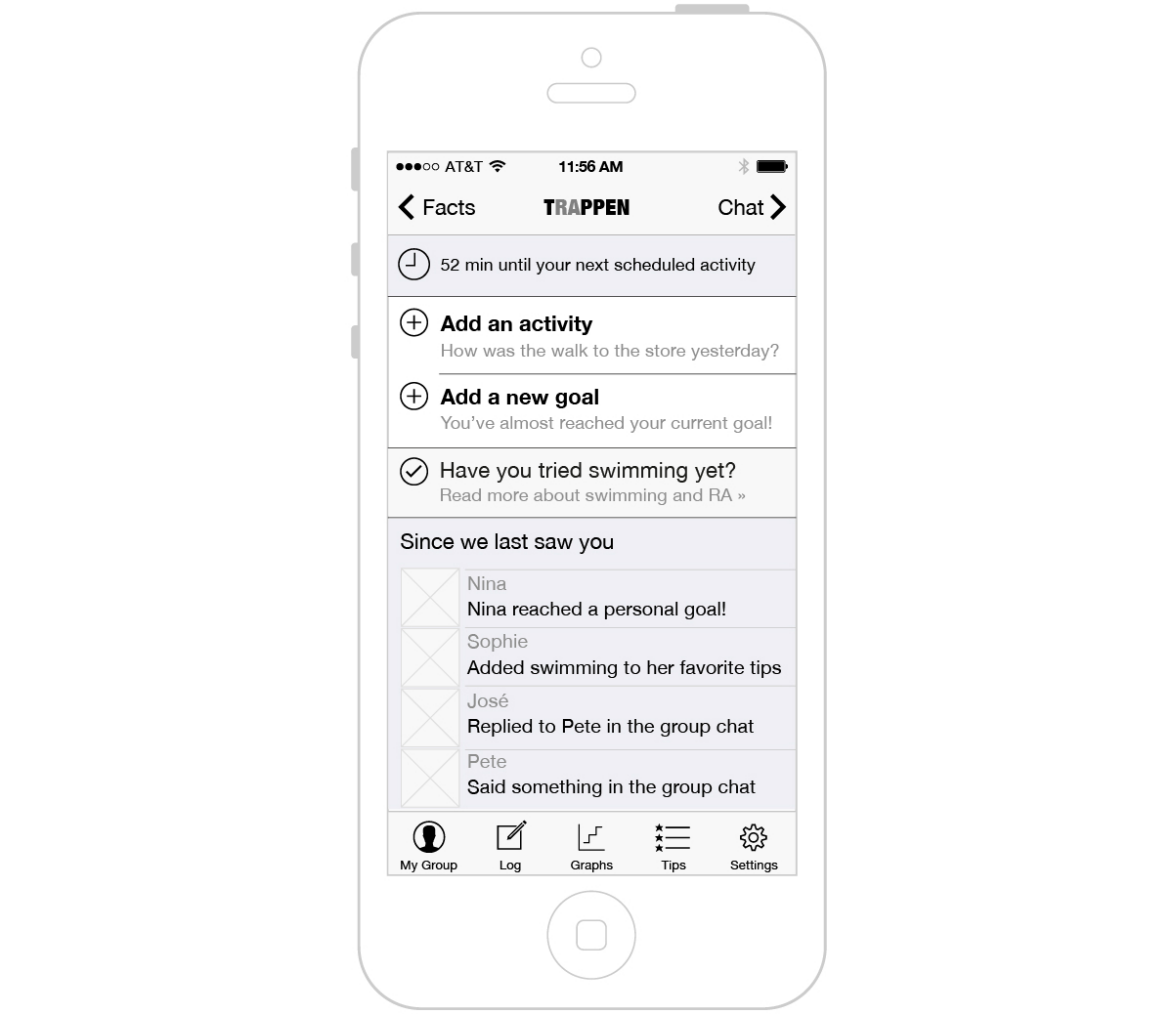
Illustration of the welcome screen of the app.

An illustration of the welcome screen of the Web-based and mobile app on a mobile phone is provided in Figure 1.

### Characteristics of the Target User Group

The users of the future app should be adults with RA, and the interface should be conducive to use by younger and older individuals. Users should have some experience with exercise and should be interested and prepared to self-manage PA with respect to individual goals and activity levels. They should also be experienced Internet users.

### Features and System Requirements

Participants’ proposals on what the app should provide, arrange, or do were compiled into clusters and reformulated as system requirements.

Table 6 presents the system requirements and the possible correlating features on the app. The presentation follows a thematic structure including system requirements and features associated with the following: (1) Recording (what data to record on the app, eg, goal setting, planning, and performance), (2) Visualization (what data to obtain from the app, eg, feedback on personal and peers’ performances and health status), (3) System Alerts (how to receive reminders or rewards from the app), (4) Social Interaction (how to provide and receive encouragement and support from individuals with RA), and (5) Facts and Information (text and links about PA in RA).

### Correlation Between System Requirements and Behavior Change Techniques

All system requirements correlated to a BCT consistent with Michie’s taxonomy [19] (Table 7). The coding resulted in 24 BCTs related to goals and planning (5 codes), feedback and monitoring (4 codes), social support (3 codes), shaping knowledge (1 code), natural consequences (2 codes), comparison of behavior (2 codes), associations (1 code), comparison of outcome (1 code), rewards and threats (3 codes), identity (1 code), and scheduled consequences (1 code).

An overview of the clustered features, system requirements, BCTs, and associated features in the future Web-based and mobile app is provided in Multimedia Appendix 1.

**Table 6:** Presentation of the system requirements and features on the app structured according to theme.

System requirements^a^		Features
**1. Recording: goal setting, planning, and self-monitoring of PA performance and health status**		
	Provide information and instructions to enable the users to set SMART (specific, measurable, accepted, realistic, time limited) goals	Features to support SMART goal setting including a calendar for PA planning
	Provide the user with the possibility to record PAs performed	A calendar feature for recording of PAs performed
	Provide the users with instructions on how to perform RA specific tests and to record the outcomes	A feature to support the performance and recording of RA specific physical tests and self-rate health tests
	Easy access for continuously review and modify set goals	A feature available on the welcome screen, to enter the goal setting screen, to review set goal
	Easy access and recording of PAs performed, rewards, and self-tests	A feature available on the welcome screen, to enter the registration screen, to record the PA performed
	Provide information and instructions on individual rewards	Features to support identification and registration of individual rewards
**2. Visualization: feedback on personal and peers’ performances and health status**		
	Provide the users with feedback on PAs performed and on health outcome	Features to provide visualized feedback on PAs performed in relation to physical fitness and health, displayed as diagrams or bars
	Provide the users with feedback and prompts, on PAs performed and on health outcome	A status indicator showing ‘my health and/or PA’ status, eg, as traffic lights or percent
	Arrange for the users to share PA performance and health status	A feature to show peer group members, name, pictures/avatars, and health status/PA goal achievement
	Visualization of planned PA	A feature to visualize the next planned PA on the welcome screen
**3. System alerts: receive reminders or rewards/punishments from app**		
	Enable the system to react to individuals not following the action plan by sending reminders to facilitate adherence to the plan	Features for the system to send reminders as text messages and emails in accordance with individual action plan
	Enable the system to give individual rewards	Features for system rewards, eg, medallions, stars
	Arrange for the system to send “punishment” if planned PA is not performed	Features for system “punishments”, eg, send an angry face
**4. Social interaction: give and receive encouragements and support from individuals with RA**		
	Enable peers to communicate with each other to help solve problematic situations, to comment on PAs performed/not performed, give specific exercise instructions, and to share own experiences of PA	A chat feature
	Enable a supportive climate for peers to: ask for and give advice on physical activity, and receive and give emotional support	Comment areas
	Provide peers with devices to send encouragements	A feature for the possibility to send encouragements/likes
**5. Facts and information: texts and links on up-to-date information about PA in RA**		
	Devices to facilitate short tips on good PAs in everyday life	A feature available on the welcome screen to present short tips on good exercises in everyday life
	Provide the users with information about positive health consequences of PA and information to reduce fear of movement	A feature to present up-to-date information on PA and access to links related to PA in RA, eg, the SRA^b^
	Provide the users with films and instructions on different PAs	A library with short films on PA on different levels
	Provide information about who will supply the app, the intention, and objectives	A section with this information

^a^What the app should provide for, arrange for, or do.

^b^Swedish Rheumatism Association.

**Table 7:** The system requirements with corresponding BCTs included in the app, presented according to the hierarchically groups described in the taxonomy.

System requirements^a^		Behavior change techniques
**Goals and planning**		
	Provide information and instructions to enable the users to SMART (specific, measurable, accepted, realistic, time limited) goal setting	Goal setting behavior—Code1.1: Set or agree on a goal in terms of the behavior to be achieved
		Action planning—Code 1.4: Prompt detailed planning of performance of the behavior
	Peers being able to communicate with each other to help solve problematic situations	Problem solving—Code 1.2: Analyze, or prompt the person to analyze, factors influencing the behavior
	Easy access for continuously review and modify set goals	Review behavior goal—Code 1.5: Review behavior goal jointly with the person and consider modifying goals
	Enable the system to react to individuals not following the action plan	Discrepancy between current behavior and goal—Code 1.6: Draw attention to discrepancy between a person’s current behavior and previously set outcome or behavioral goals or action plans
**Feedback and monitoring**		
	Provide the users with feedback on PA performed	Feedback on behavior—Code 2.2: Monitor and provide informative or evaluative feedback on performance of the behavior
	Provide the user with the possibility to record physical activities performed	Self-monitoring of behavior—Code 2.3: Establish a method for the person to monitor and record their behavior
	Provide the users with instructions to enable the users to perform RA-specific tests and to record their performances	Self-monitoring of outcome—Code 2.4: Establish a method for the person to monitor and record the outcome of their behavior
	Easy access and recording of PAs performed, rewards and self-rate health tests	Self-monitoring of behavior (2.3); Self-monitoring of outcome (2.4)
	Provide the users with feedback on health outcome	Feedback on outcome of behavior—Code 2.7: Monitor and provide feedback on the outcome of performance of the behavior
**Social support**		
	Facilitate for peers to comment on PAs performed/not performed	Social support (unspecified)—Code 3.1: Advise on, arrange, or provide social support or non-contingent praise or reward
	Enable a supportive climate for peers to ask for and give advice on PA	Social support (practical)—Code 3.2: Advise on, arrange, or provide practical help
	Enable a supportive climate for peers to receive and give emotional support	Social support (emotional)—Code 3.3 Advise on, arrange, or provide emotional social support
**Shaping knowledge**		
	Peers being able to give specific descriptions on how to perform exercises	Instructions on how to perform a behavior—Code 4.1: Advise or agree on how to perform the behavior (includes skills training)
	Devices to facilitate the provision of short tips on good exercises in everyday life	Instructions on how to perform a behavior (4.1); Also coded as Prompts/cues (7.1)
**Natural consequences**		
	Provide the users with information about positive health consequences of PA	Information about health consequences—Code 5.1: Provide information about health consequences of performing the behavior
	Provide information to reduce fear of movement	Information about emotional consequences—Code 5.6: Provide information about emotional consequences of performing the behavior
**Comparison of behavior**		
	Provide the users with films and instructions on different PAs	Demonstration of the behavior—Code 6.1: Provide an observable sample of the performance of the behavior, for the person to aspire to or imitate
	Arrange for the users to share PA performance and health status	Social comparison—Code 6.2: Draw attention to others’ performances to allow comparison with own performance.

**Associations**		
	Provide the users with prompts to stimulate PA	Prompts/cues—Code 7.1: Introduce or define environmental or social stimulus with the purpose of prompting or cueing the behavior
	Visualization of planned PA	
	Enable the system to send reminders to facilitate adherence to the action plan	
	Devices to facilitate the provision of short tips on good exercises in everyday life	Prompts/cues (7.1); Also coded as Instructions on how to perform a behavior (4.1)
**Comparison of outcome**		
	Provide information about who will supply the app, the intention, and objectives	Credible source—Code 9.1: Present verbal or visual communication from a credible source
**Rewards and threats**		
	Provide information and instructions on individual rewards	Material incentive—Code 10.1: Inform that valued objects will be delivered if effort/progress in performing the behavior
	Provide peers with devices to send encouragements	Social reward—Code 10.4: Arrange verbal or non-verbal reward if there has been effort and/or progress in performing the behavior
	Enable the system to give individual rewards	Non-specific incentive—Code 10.6: Inform that a reward will be delivered if and only if there has been effort and/or progress in performing the behavior
**Identity**		
	Enable peers to share own experiences of PA	Identification of self as a role model—Code 13.1: Inform that one’s own behavior may be an example to others
**Scheduled consequences**		
	Arrange for the system to send “punishment” if planned PA is not performed	Punishment—Code 14.2: Arrange for aversive consequence

^a^What the app should provide for, arrange for, or do.

## Discussion

### Principal Findings

The co-design participants agreed that features enabling self-monitoring and peer support were important to self-manage and maintain physically active lifestyles in individuals with RA and that such features should be included in the future Web-based and mobile app.

The app should provide information and instructions to enable SMART (specific, measurable, accepted, realistic, time limited) goal setting, activity planning and recording of PA performance and progress. The participants suggested that these requirements be integrated into a calendar feature. Recent research has identified a calendar feature as the most useful tool in increasing PA in the general adult population [31]. Furthermore, self-monitoring of PA and goal setting is known to improve the efficacy of PA self-management interventions [20,32].

The app should also provide visual feedback on PA performance and health outcomes. It was suggested that these features be displayed in diagrams or as health status indicators. Feedback on PA performance is associated with increased effects of interventions [20], which is consistent with health behavior change theories describing feedback important for motivating, reinforcing, and guiding individuals [17]. The feedback may take a variety of forms, for example, verbal print communication or telephone counseling. Our participants requested visualized feedback. However, the difficulty of designing diagrams to encourage PA was highlighted. Research is limited in this area; however, how feedback is provided visually has previously been demonstrated to be important [33].

Participants also agreed that peer support was important in maintaining a physically active lifestyle. It was suggested that the future app should include small communities of peers consisting of 10 individuals with RA, who would be expected to provide advice, encouragement, and help in providing solutions to problematic situations. The majority of self-management programs for individuals with arthritis include online or face-to-face support from health care providers [14]. However, the Arthritis Self-Management Program [34,35] is based on small communities of patients moderated by a trained peer. The program has been demonstrated to have a positive impact on disease symptoms, general health, and health care consumption. We hypothesize that the knowledge and expertise of peers with RA is one effective strategy for facilitating the maintenance of PA. However, future studies are required to test this hypothesis.

Peer support was one of several features that were perceived as unique and of significant importance for supporting the maintenance of PA in individuals with RA. The importance of easily modifying established goals based on variations in the disease course was also emphasized by the participants as a specific requirement for the population of RA. This was perceived to be crucial to encourage and self-manage PA during arthritis episodes. Our participants also wanted to monitor and follow up on health status. To support this desire, written and visual instructions on how to perform PA and health tests specific to RA were suggested to be included in the app.

Programs supporting different health behaviors are growing in number. Health care-delivered Internet programs/interventions targeting PA behavior have been reported effective in adult populations as well as in populations with different diseases and disabilities [15,32,36]. Similar to these interventions, the future app will include peer support, self-monitoring (eg, goal setting), and feedback. In addition, there is a growing number of commercial mobile apps available targeting PA. However, a recent review concluded that there is still a lack of mobile apps that incorporate theories on behavior change [37]. Most PA apps include predesigned exercise plans, exercise instructions and registrations, and lack the inclusion of social interaction and personal goal setting [37]. The future Web-based and mobile app will differ from existing mobile apps by inclusion of features deliberately derived from theories on behavior change. It will also be adapted to the specific needs of the population of RA.

The importance of social support and self-monitoring when self-managing PA is described in learning and behavior change theories [12,17,38]. Self-monitoring is a method used to self-regulate a behavior. Self-regulation skills, defined as an individual’s ability to endure short-term negative outcomes to achieve a long-term goal [38], are described in SCT [12]. The participants in our study emphasized the importance of features supporting self-regulation, such as setting and reviewing goals, planning and recording PA performance and receiving feedback. Social interaction is also described in SCT as being important for self-management. Social interaction may be part of self-regulation when peers who encourage behavioral control are accessible [17,38]. Social interaction also provides observational learning or peer modeling, another main construct in SCT [38]. Features to show the peer-group members’ PA and general health status and chats or comment areas for users to share experiences may be examples of the aforementioned SCT construct.

### Limitations

This study has some limitations. Although the participants diagnosed with RA (n=5) varied with respect to age, gender, years living with the disease, and PA habits, the small number reduced the variations in proposals and preferences. The limited number of participants was due to the requirement of creating a convenient work group (N=10). The creation of two separate work groups was also considered; however, it was not possible due to economic and time limits. However, the results from the first step of the co-design process [29] were the starting point for the workshops; thus, they may have added to variations in proposals considered in this study. Nevertheless, this study may lack a description of features essential for the target users of the future app. In summary, the generalizability of the results from this study to the target user group of the future app remains unknown. In the next step of the development process, it is therefore important to test the first versions of the Web-based and mobile app in iterative cycles with representatives from the target-user group.

Another limitation is associated with the participants’ experiences of mobile apps to support their PA. Only 40% of the participants with RA reported having tried similar apps, which may have hampered discussions on features of our future app. However, to facilitate the discussions during the workshops, the features were discussed as “I want to be able to...”. These proposals were then translated into possible features on a computer or mobile phone by the moderator or the other participants.

### Strengths

One strength of this study is the use of a BCT taxonomy to describe the intervention. Our results show that the system requirements were possible to associate with theoretically derived BCTs using the taxonomy established by Michie et al [19]. Previous studies reporting on the use of BCT taxonomy have used it to describe and compare intervention features [39-41]. We experienced a few obstacles when using the taxonomy. The features had to be clearly described to fit to a single corresponding BCT. The video recordings enabled us to obtain that additional data. There is also an interpretation component to be aware of when using the taxonomy. To improve the credibility of the coding in this study, 3 researchers were involved in the coding, which enabled discussion of disparities and how to interpret the definitions in the taxonomy. Coding the system requirements based on the taxonomy provided an additional description of the features. A future challenge for the development of mobile apps aimed for behavior change is the question of how to combine the inclusion of several BCTs that seem to be preferable to enable behavior change, and a user-friendly and simple application. Hence, in the future, we need to identify how many and which BCTs to include in our future app.

Another strength is the use of co-design, that is, active involvement of users throughout the development process. Involvement of users in development of health services has been described as significant for the viability, usability, and effectiveness of services [21,42]. We propose that this method of co-designing decreases the interpretation of the users’ requirements and preferences due to the ability to discuss the uncertain matters during the production of the requirement specification. However, this method is also time consuming and challenging with regard to, for example, the importance of merging the different perspectives and the difficulty involved in managing the professional role. The opportunities and challenges of co-design as performed in this study will be further explored and reported elsewhere.

### Conclusions

This study provides basic data on a requirement specification of an eHealth service to support the maintenance of physical activity adapted to the specific needs of individuals with rheumatoid arthritis. The participants agreed on the importance of including features for self-monitoring and peer support in maintaining a physically active lifestyle. The results are consistent with learning and behavior change theories, which describe physical activity as a complex behavior determined by personal, behavioral, and environmental factors. The system requirements correlated with BCTs, which may improve the possibility of replicating and evaluating the future Web-based and mobile app and, furthermore, enables the identification of how many and which BCTs to include, thereby advancing our knowledge of health behavior change.

The use of co-design in this study is based on our assumption that people living with a chronic disease, such as RA, have experiences and knowledge that can improve health care services. There may also be an intrinsic value for people with RA knowing that the app was developed by peers. By involving the users in the requirement specification, we hope to be able to further refine the features according to the users’ needs. In the next step of development, the first version of the app will be produced and tested by new users.

## Multimedia Appendix 1

Overview of the clustered features, system requirements, BCTs, and associated features on the app, thematically grouped based on associations with Recording, Visualization, System Alerts, Social Interaction, and Facts and Information.

## Abbreviations

BCT: behavior change technique
PA: physical activity
RA: rheumatoid arthritis
SCT: social cognitive theory
SRA: Swedish rheumatism association
