# Supplementary material for: Development of a Web-Based and Mobile App to Support Physical Activity in Individuals With Rheumatoid Arthritis: Results From the Second Step of a Co-Design Process
Source: JMIR Res Protoc. 2015 Feb 9;4(1):e22. doi: 10.2196/resprot.3795 (PMC4342685; doi:10.2196/resprot.3795)
Supplement: Supplementary file 1 [file resprot_v4i1e22_app1.pdf]

1. Recording: goal setting, planning and self-monitoring of physical activity performance and health status

Features [a]	System requirements [b]	BCT [c]	The App
• Setting interim goals to help achieve long term goals<br>• Individual long term goals<br>• SMART goal setting	Provide information and instructions to enable the users to SMART (specific, measurable, accepted, realistic, time limited) goal set	**Goal-setting behavior**<br>Code1.1: Set or agree on a goal in terms of the behavior to be achieved	• Features to support SMART goal setting including a calendar for physical activity planning
• Activity planning<br>• Individualized exercise program to help achieve goals<br>• Help with and follow-up of activity planning		**Action planning**<br>Code 1.4: Prompt detailed planning of performance of the behavior	
• Record exercises performed	Provide the user with the possibility to record physical activities performed	**Self-monitoring of behavior**<br>Code 2.3: Establish a method for the individual to monitor and record his or her behavior	• A calendar feature for recording of physical activities performed
• Record well-being<br>• Perform and record the results of RA-specific physical tests	Provide the users with instructions to enable the users to perform RA-specific tests and record the outcome	**Self-monitoring of outcome**<br>Code 2.4: Establish a method for the individual to monitor and record the outcome of his or her behavior	• A feature to support the performance and recording of RA- specific physical tests and self-rate health tests
• Change goal according to well-being<br>• Change goal if set goal is not achieved	Easy access for continuously reviewing and modifying set goals	**Review behavior goal**<br>Code 1.5: Review behavior goal jointly with the individual and consider modifying goals	• A feature available on the welcome screen to review set goal
• Record exercise in the app same day<br>• Automatic registration of activities available in the app	Easy access for recording of physical activities performed	**Self-monitoring of behavior** (2.3)<br>**Self-monitoring of outcome** (2.4)	• A feature available on the welcome screen to record the physical activity performed
• Determine reward if goal is achieved	Provide information and instructions on how to determine individual rewards	**Material incentive**<br>Code 10.1: Inform that valued objects will be delivered if effort/progress in performing the behavior	• Features to support identification and recording of individual rewards

[a] The participants' proposals on features retrieved from the on-line notice board, [b] What the app should provide, arrange or do, [c] BCTs as described and classified by Michie et al.,(2013)

2. Visualization: feedback personal and peers physical activity performances and health status.

Features [a]	System requirements [b]	BCT [c]	The App
• To learn from own exercise behavior to reduce risk to fall back into old habits<br>• Show diagrams of physical activity goal achievement<br>• Show frequency of exercise	Provide the users with feedback on physical activities performed	**Feedback on behavior**<br>Code 2.2: Monitor and provide informative or evaluative feedback on performance of the behavior	• Features to provide visualized feedback on physical activities performed in relation to physical fitness and health and that are displayed as diagrams or bars
• Show physical and mental well-being<br>• Being able to see what makes the exercise plan work or not at different times<br>• Visualize how exercise affects my health<br>• Self-rate current well-being to see change over time	Provide the users with feedback on health outcome	**Feedback on outcome of behavior**<br>Code 2.7: Monitor and provide feedback on the outcome of performance of the behavior	
• Activity indicator based on self-tests	Provide the users with feedback on physical activities performed	**Feedback on behavior** (2.2)	• A status indicator showing 'my health and/or physical activity ' status, eg., as traffic lights or percent
• Health indicator based on self-tests	Provide the users with feedback on health outcome	**Feedback on outcome of behavior** (2.7)	
• Aim for indicator to show green light	Provide the users with prompts to stimulate physical activity	**Prompts/cues**<br>Code 7.1: Introduce or define environmental or social stimulus with the purpose of prompting or cueing the behavior	
• Display status of group members	Arrange for the users to share physical activity performance and health status	**Social comparison**<br>Code 6.2: Draw attention to others' performances to enable comparison with the individuals' own performance.	• A feature to show peer group members, name, pictures/avatars and status in relation to physical activity goal achievement and physical activity performed
• Display planned exercise	Visualization of planned physical activity	**Prompts/cues** (7.1)	• A feature to visualize the next planned physical activity on the welcome screen

[a] The participants' proposals on features retrieved from the on-line notice board, [b] What the app should provide, arrange or do, [c] BCTs as described and classified by Michie et al.,(2013)

3. System alerts: receive reminders or rewards/punishments from the app

Features [a]	System requirements [b]	BCT [c]	The App
• Alert when planned exercise is not performed	Enable the system to react to individuals not following the action plan	**Discrepancy between current behavior and goal**<br>Code 1.6: Draw attention to discrepancies between an individuals' current behavior and previously established outcome, behavioral goals or action plans<br><br>**Prompts/cues** (7.1)	• Features for the system to send reminders as text messages and e-mails based on the individual's action plan
• Reminder in advance to exercise	Enable the system to send reminders to facilitate adherence to the action plan		
• Rewards at goal achievement or after reaching predetermined level of exercise intensity<br>• Receiving rewards from the system	Enable the system to provide individual rewards	**Non-specific incentive**<br>Code 10.6: Inform that a reward will be delivered if and only if there has been effort and/or progress in performing the behavior	• Features for system rewards, eg., medallions, stars
• Receive negative response when not performing planned exercise	Arrange for the system to send ''punishment '' if planned physical activity is not performed	**Punishment**<br>Code 14.2: Arrange for aversive consequence	• Features for system punishments, eg., send an angry face

[a] The participants' proposals on features retrieved from the on-line notice board, [b] What the app should provide, arrange or do, [c] BCTs as described and classified by Michie et al.,(2013)

4. Social interaction: give and receive encouragements and support from individuals with RA.

Features [a]	System requirements [b]	BCT [c]	The app
• Receive questions about previously performed exercises<br>• Receive specific suggestions for alternative exercise when in pain<br>• Receive help with self-analysis to choose appropriate level of exercise<br>• Letting off steam/vent with peers	Peers being able to communicate with each other to help solve problematic situations	**Problem solving**<br>Code 1.2: Analyze, or prompt the person to analyze, factors influencing the behavior	• A chat feature<br>• Comment areas
• Ask for encouragement<br>• Get reactions from peers when not exercising	Peers being able to comment on physical activities performed/not performed	**Social support (unspecified)**<br>Code 3.1: Advise on, arrange or provide social support or non-contingent praise or reward	
• Receive tips and advice on exercise<br>• Ask for advice	Enable a supportive climate for peers to ask for and provide advice on physical activity	**Social support (practical)**<br>Code 3.2: Advise on, arrange or provide practical help	
• Establish a relationship with the peer group<br>• See how my peers are doing<br>• Give or receive encouragement	Enable a supportive climate for peers to receive and provide emotional support	**Social support (emotional)**<br>Code 3.3 Advise on, arrange or provide emotional social support	
• Specific exercise suggestions<br>• Learn how peers exercise from their own accounts	Peers being able to provide specific exercise instructions	**Instructions on how to perform a behavior**<br>Code 4.1: Advise or agree on how to perform the behavior (includes skills training)	
• Recommend favorite exercise to peers<br>• Give advice to peers in similar situations	Enable peers to share their own experiences of physical activity	**Identification of self as a role model**<br>Code 13.1: Inform that one's own behavior may be an example to others	
• Receive encouragement when performed exercise or reached goals	Provide peers with devices to send encouragements	**Social reward**<br>Code 10.4: Arrange verbal or non-verbal reward if there has been effort and/or progress in performing the behavior	• A feature for the possibility to send encouragements/likes

[a] The participants' proposals on features retrieved from the on-line notice board, [b] What the app should provide, arrange or do, [c] BCTs as described and classified by Michie et al.,(2013)

5. Facts and information: texts and links on physical activity in RA

Features [a]	System requirements [b]	BCT [c]	The app
• Tips on good exercises in everyday life	Devices to facilitate short tips on good physical activities in everyday life	**Instructions on how to perform a behavior** (4.1)<br>**Prompts/cues** (7.1)	• A feature available on the welcome screen to present short tips on good physical activities in everyday life
• Information about positive health consequences of physical activity	Provide the users with information on the positive health consequences of physical activity	**Information about health consequences**<br>Code 5.1: Provide information about health consequences of performing the behavior	• A feature to present up to date information on physical activity<br>• Access to links related to physical activity in RA, eg. the SRA [d]
• Reduce fear of movement	Provide information to reduce fear of movement	**Information about emotional consequences**<br>Code 5.6: Provide information about emotional consequences of performing the behavior	
• Library of exercise films for different levels	Provide the users with films and instructions on different physical activities	**Demonstration of the behavior**<br>Code 6.1: Provide an observable sample of the performance of the behavior, for the person to aspire to or imitate	• A library with short films on physical activity on different levels.
• Receive information from a reliable source	Provide information on who will supply the WeMApp and the intention and objectives of the WeMApp	**Credible source**<br>Code 9.1: Present verbal or visual communication from a credible source	• A section containing this information

[a] The participants' proposals on features retrieved from the on-line notice board, [b] What the app should provide, arrange or do, [c] BCTs as described and classified by Michie et al.,(2013), [d] Swedish Rheumatism Association
